# Sleep position and sleep apnea in newborns

**DOI:** 10.1007/s44470-026-00218-0

**Published:** 2026-11-03

**Authors:** Lisa B. Matlen, Ronald D. Chervin, John D. E. Barks, Fauziya Hassan, Stephanie A. Eyerly-Webb, Brandon G. Rocque, Ellen Bendel-Stenzel, Robin M. Lloyd, John E. Pascoe, Narong Simakajornboon, Jagruti Anadkat, Stephen V. Gliske, Cindy Jon, Danna Tauber, Harlan McCaffery, Nico Kaciroti, Stephanie M. D. Rau, Renée A. Shellhaas

**Affiliations:** 1 Department of Pediatrics, University of Michigan, Ann Arbor, MI USA; 2 Department of Neurology and Sleep Disorders Center, University of Michigan, Ann Arbor, MI USA; 3 Midwest Fetal Care Center, Children’s Minnesota, Minneapolis, MN USA; 4 Department of Neurosurgery, University of Alabama at Birmingham, Birmingham, AL USA; 5 Department of Neonatology, Mayo Clinic Children’s, Rochester, MN USA; 6 Department of Pediatrics, Mayo Clinic Children’s, Rochester, MN USA; 7 Division of Pulmonary and Sleep Medicine, Cincinnati Children’s Hospital Medical Center, Cincinnati, OH USA; 8 Department of Pediatrics, University of Cincinnati College of Medicine, Cincinnati, OH USA; 9 Division of Pulmonary and Sleep Medicine, Department of Pediatrics, Cincinnati Children’s Hospital Medical Center, Cincinnati, OH USA; 10 Department of Pediatrics, Washington University in St. Louis, St. Louis, MO USA; 11 Department of Neurosurgery, University of Nebraska Medical Center, Omaha, NE USA; 12 Department of Pediatrics, UTHealth-Houston, Houston, TX USA; 13 Division of Pulmonary Sleep Medicine, Department of Pediatrics, Children’s Hospital of Philadelphia, Philadelphia, PA USA; 14 Department of Biostatistics, University of Michigan, Ann Arbor, MI USA; 15 Department of Neurology, Washington University, St. Louis, MO USA

**Keywords:** Neonate, Polysomnogram, Sleep apnea, NICU, Myelomeningocele, Prematurity

## Abstract

**Purpose:**

Public health measures recommend that babies sleep supine, but at all other times of life this can worsen sleep apnea. To examine associations between infant sleep position and the apnea–hypopnea index (AHI), we studied polysomnograms of neonates with repaired myelomeningocele, who are often positioned nonsupine during wound healing, and of neonates without congenital anomalies who were recovering in the neonatal intensive care unit (NICU).

**Methods:**

We evaluated sleep apnea severity, as reflected by the AHI, in different positions as recorded during polysomnograms from three cohort studies of infants in NICUs. Participants included 173 infants with myelomeningocele (multiple hospitals) and 88 NICU recovery infants across two single-center cohorts. The myelomeningocele cohort was subdivided by repair type (postnatal, open fetal, and fetoscopic surgery groups). We evaluated AHI in different sleep positions using generalized estimating equations to account for repeated measures.

**Results:**

Among 261 neonates, AHI was similar between supine and nonsupine positions in the NICU recovery, postnatal myelomeningocele, and open fetal myelomeningocele groups (adjusted analyses, all *p* > 0.33). In contrast, the fetoscopic myelomeningocele group had substantially lower AHI when supine (7.94, 95% CI 4.95–12.75) compared to nonsupine (22.1, 95% CI 14.02–35.01; *p* < 0.001).

**Conclusion:**

Surprisingly, sleep apnea in neonates requiring NICU care may not be exacerbated by supine sleep, with possible exceptions in some specific conditions. Although we did not study healthy newborns, we speculate that obligate nasal breathing in neonates may protect against worsening of sleep apnea when supine. “Back to Sleep” remains life-saving and appears unlikely to increase sleep apnea risk.

**Brief summary:**

**Current knowledge/study rationale:**

Public health measures to prevent SIDS recommend that babies sleep supine. The degree to which sleep position influences sleep apnea in infants is unclear. We had a unique opportunity to study the position dependence of sleep apnea for neonates in the NICU setting, who sometimes sleep in nonsupine positions. As studies at older ages widely show sleep apnea to be worse supine than nonsupine, we hypothesized that we would find the same in neonates.

**Study impact:**

Study results suggest that sleep apnea severity in neonates who require NICU care is not exacerbated by supine sleep. “Back to Sleep” remains a life-saving intervention for infants upon discharge to home and, moreover, does not appear to increase risk for sleep apnea.

**Graphical Abstract:**

**Figure:**
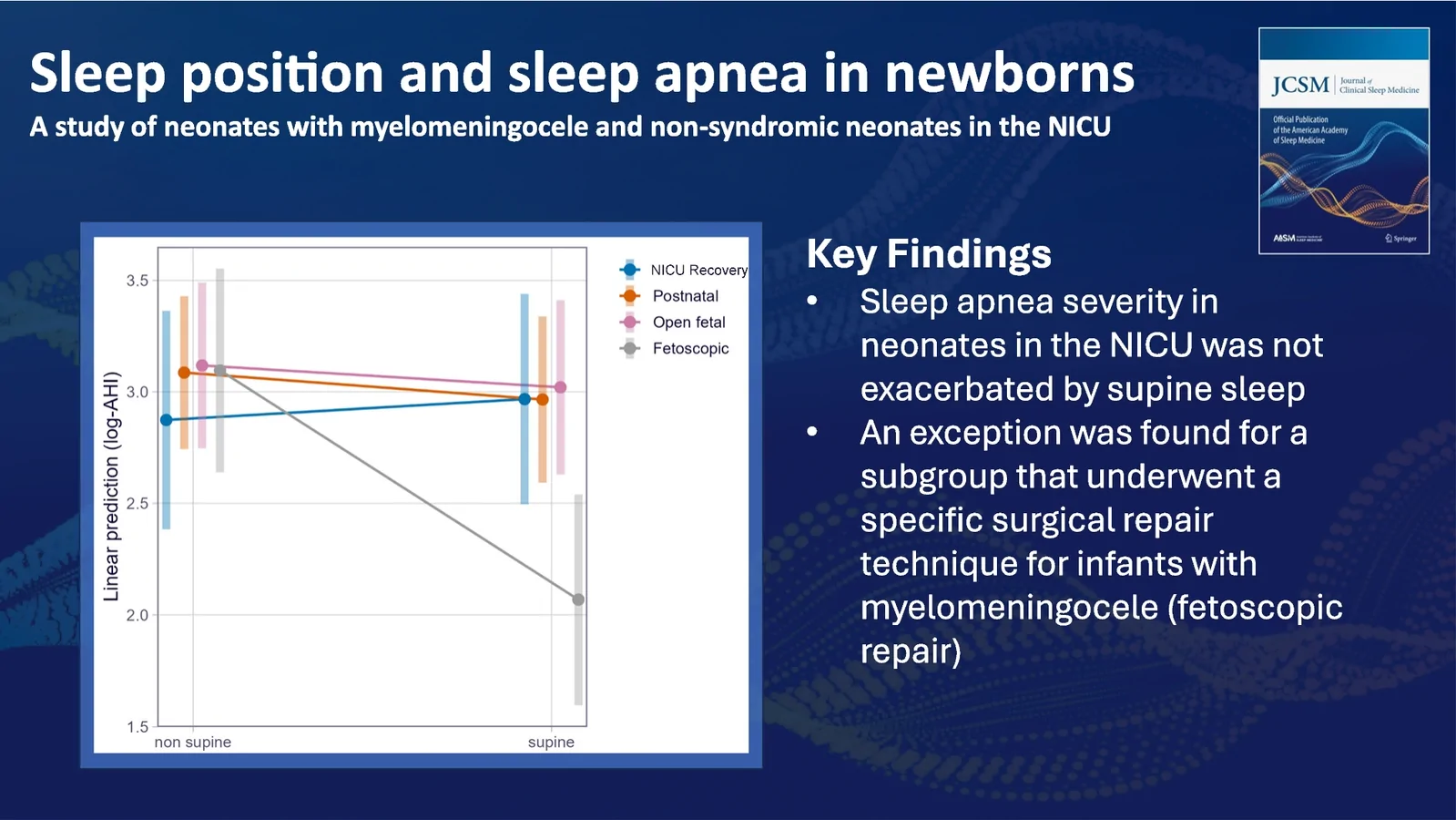

**Supplementary Information:**

The online version contains supplementary material available at https://doi.org/10.1007/s44470-026-00218-0.

## Introduction

The early 1990 s public safety campaign “Back to Sleep” reduced the risk of sudden infant death syndrome (SIDS) [1, 2], thereby justifying supine sleep as the standard recommendation for all infants. Yet, in older children and adults, supine sleep is associated with increased severity of sleep apnea [3–5]. Thus, while clearly associated with reduced risk of SIDS, the “Back to Sleep” guidance did not address questions about whether infants are susceptible to supine position-dependent worsening of sleep apnea.

Few studies have evaluated positional sleep apnea in infants, though some suggest that extreme cardiovascular events may occur independent of sleep position [6]. Since a randomized clinical trial of different sleep positions would raise safety concerns, we sought to leverage observational data from selected inpatient newborn groups to evaluate the hypothesis that sleep apnea severity, as quantified by apnea–hypopnea index (AHI), is worse in the supine position than nonsupine positions for newborn infants at high risk of sleep apnea.

Some exceptions arise to strict supine positioning in the neonatal intensive care unit (NICU) setting. For example, infants with myelomeningocele are often side-lying or even prone to minimize pressure on their surgical site (although practices vary across institutions). Additionally, some preterm infants sleep in nonsupine positions due to pulmonary disease. We had a unique opportunity to assess for associations between sleep position and AHI using polysomnographic data recorded for research purposes from neonates with myelomeningocele and from neonates without congenital anomalies who required NICU care.

## Methods

Each of the cohort studies was approved by the Institutional Review Board at the University of Michigan. Written informed consent was obtained from a parent of every enrolled infant.

### Participants

This is a secondary analysis of three cohort studies (Table 1): (1) a multicenter study of sleep apnea in infants with myelomeningocele (MMC, *N* = 173) [7], (2) a single-center study of infants who were late-preterm to term without congenital anomalies and required NICU critical care but were in the recovery phase (“NICU acoustics” study, *N* = 47) [8, 9], and (3) a separate single-center study of infants without congenital anomalies who were late-preterm to term and required NICU care (“technology development” study, *N* = 39; previously unpublished).

**Table 1:** Characteristics of three neonatal polysomnography cohort studies (overall *N* = 261)

	Myelomeningocele (MMC) (*N* = 173) [7]	NICU Acoustics (*N* = 49) [8, 9]	Technology Development (*N* = 39)
		NICU Recovery	
Study design	Multicenter	Single center	Single center
Enrollment dates	July 2020–April 2024	February 2015–July 2017	September 2021–December 2024
PSG duration	10 h	12 h	12 h
Inclusion criteria	Myelomeningocele: • Fetal closure (*N* = 90; 56 open fetal surgery, 34 fetoscopic surgery) • Postnatal closure (*N* = 83) PSGs performed when: • Medically stable following surgical closure • No acute infection or recent/urgently planned surgery (e.g., shunt placement) • > 24 h after any surgery • > 35 weeks postmenstrual age • Prior to NICU discharge	• Gestational age at birth ≥ 33–41 weeks • NICU admission • Stable in open crib • PSG performed at 33–43 weeks postmenstrual age	• Gestational age at birth ≥ 33–41 weeks • NICU admission • Stable in open crib • PSG performed at 33–43 weeks postmenstrual age
Exclusion criteria	• < 30 weeks’ gestation at birth • Congenital anomalies that would predispose to sleep apnea (e.g., micrognathia) • Confirmed or suspected genetic syndromes that would affect developmental trajectory	• Congenital brain abnormalities • Known or suspected genetic syndromes that could result in cerebral dysfunction • Severe encephalopathy that precluded sleep–wake cycling • Significant airway abnormalities likely to result in sleep apnea • Abnormal brainstem auditory evoked responses	• Congenital brain abnormalities • Known or suspected genetic syndromes that could result in cerebral dysfunction • Severe encephalopathy that precluded sleep–wake cycling • Significant airway abnormalities likely to result in sleep apnea • Congenital skin conditions or skin conditions that the investigator or clinical team assessed put them at greater risk

The NICU acoustics study examined electroencephalogram (EEG) changes in response to maternal voice exposure during sleep [8, 9]. The technology development study examined automated detection of sleep staging via EEG parameters. The NICU acoustics and technology development studies shared a single-center setting, comparable inclusion criteria (no congenital anomalies, gestational age 33–41 weeks, NICU admission, stable in an open crib), similar exclusion criteria (congenital brain abnormalities, significant airway abnormalities, suspected genetic syndromes, severe encephalopathy), and identical 12-h polysomnographic recording protocols; they were therefore pooled into a single “NICU recovery” cohort for the present position-effect analyses. Thus, the four participant groups for the current analyses were a single “NICU recovery” cohort and three MMC surgical groups distinguished by the timing and type of their myelomeningocele repair—postnatal repair, open fetal repair, or fetoscopic repair. Surgical timing and technique (e.g., postnatal vs. open maternal–fetal surgery vs. fetoscopic MMC closure) was determined by the local clinical teams and was not influenced by study participation. Similarly, clinical protocols for sleep positioning were determined by the clinical teams.

### Measurements

Clinical and demographic data were extracted from the medical records. Each infant underwent a polysomnogram at the bedside prior to hospital discharge. A registered polysomnographic technologist remained at the bedside throughout the study and recorded the infants’ sleep positions. Standard polysomnography data included at least a 6-channel neonatal-montage [10, 11] electroencephalogram (EEG), bilateral electrooculogram (EOG), chin surface electromyogram (EMG), nasal pressure airflow, nasal/oral airflow (thermocouples), chest and abdominal excursion (inductance plethysmography), snore sensor, oxygen saturation, transcutaneous CO_2_, electrocardiogram, bilateral anterior tibialis surface EMG, and digital video. The MMC cohort’s polysomnograms were designed to have durations of 10 h, as analyses from the previous groups had suggested that 12-h polysomnograms (as in NICU recovery) did not provide added advantage. Recording parameters were otherwise the same across cohorts.

Polysomnograms for the MMC study were centrally scored and interpreted by one experienced sleep technologist and two board-certified pediatric sleep physicians, who reviewed separately and, in the case of ambiguity, formed a consensus on interpretation [7]. Polysomnograms for the NICU recovery cohort were scored by the same experienced sleep technologist who scored the MMC study polysomnograms and reviewed by one board-certified pediatric sleep physician. Positions were annotated directly on the records by the bedside sleep technologists. Procedures and scoring followed contemporary published standards as in the American Academy of Sleep Medicine (AASM) scoring manual, as supported by the original infant manual from Anders et al. 1971 [12]. There were no significant differences between the contemporaneous AASM scoring manual’s definitions of respiratory events between 2015 and 2018, the latest used version. In summary, all scoring included two-breath-length minimum durations for apneas and hypopneas, followed by 3% oxygen desaturations or arousals for hypopneas and central apneas. Central apneas may be scored with 20-s event duration and bradycardia.

### Analysis

Descriptive statistics were used to summarize total sleep time (TST) and sleep apnea indices including AHI, obstructive apnea index (OAI), central apnea index (CAI), and hypopnea index (HI) by sleep position, cohort, and MMC surgery group. Continuous variables were summarized as median (Q1, Q3) given that sleep apnea indices and TST were not normally distributed. Sleep apnea indices were set to missing when TST in a given position was less than 60 min. Some neonates had prone sleep recorded at one center within the MMC cohort, but the prone data were too sparse for separate analysis; prone position observations were therefore combined with lateral position into a nonsupine sleep position category. Between-group comparisons across studies, surgery groups, or sites were conducted using Kruskal–Wallis tests, with two-sided *p* values used to assess statistical significance.

We used a linear mixed model to determine whether AHI varied with sleep position (supine vs. nonsupine) and whether this relationship differed between the MMC and NICU recovery cohorts. Because the population distribution of AHI values tends to be skewed and linear regression relies on the assumption of normally distributed residuals, the outcome was log-transformed prior to analysis; all results are reported on the original scale as estimated geometric means with 95% confidence intervals. The model included gestational age (GA) as a covariate and a statistical interaction term between sleep position and participant cohort or MMC surgical group to test whether the effect of position varied by cohort or MMC surgical group.

Some newborns contributed observations in both supine and nonsupine sleep positions while others were observed in only one position, so the model included random intercepts at the participant level to account for this within-person structure, as well as at the site level to account for enrollment across multiple clinical sites. The total analytic sample included 346 observations from 255 participants (six of the 261 enrolled neonates contributed no observations to the mixed model because none of their sleep periods met the 60-min minimum threshold in either position).

The study had 94% statistical power to detect a clinically meaningful ≥ 10-point difference in AHI by sleep position and 80% power to detect an AHI difference as small as 8.3 points.

All analyses were conducted in R version 4.5.1 using the lme4 and emmeans packages.

## Results

The polysomnograms of 261 neonates were analyzed across the MMC and NICU recovery cohorts (Table 2). In the MMC cohort, 167/173 infants met inclusion criteria for at least 60 min of recorded sleep in at least one position (87 infants had ≥ 60 min supine and 133 infants had ≥ 60 min of sleep in nonsupine positions). The distribution of sleep positions varied significantly across the nine study centers in the MMC study (*p* < 0.001; Supplementary Table S1). In the NICU recovery cohort, 87/88 infants had ≥ 60 min of supine sleep and 39/88 infants ≥ 60 min of nonsupine sleep. More TST (*p* < 0.001) was captured in the NICU recovery cohort as the polysomnogram recordings were longer (516 min, IQR 431–609.6) than the MMC cohort (397 min, IQR 323–440.5).

**Table 2:** Demographic and sleep position characteristics of the MMC and NICU recovery cohorts

Variable	MMC (*N* = 173)	NICU recovery (*N* = 88)	Total (*N* = 261)	*p* value
Gestational age (weeks)	37.0 (34.7, 38.0)	34.1 (33.3, 35.9)	36.0 (34.0, 37.6)	**< 0.001**
Age at NICU PSG (weeks)	1.4 (0.9, 2.6)	1.0 (0.6, 2.7)	1.3 (0.7, 2.6)	0.14
Postmenstrual age at PSG (weeks)	38.4 (37.0, 39.7)	36.1 (35.0, 37.7)	37.6 (36.1, 39.6)	**< 0.001**
Birth weight (grams)	2840.0 (2370.0, 3200.0)	2290.0 (1838.8, 2650.0)	2630.0 (2120.0, 3120.0)	**< 0.001**
Sex: female	80 (46.2%)	53 (60.2%)	133 (51.0%)	**0.033**
Supine time/TST (%)	22.7 (0.0, 69.4)	93.4 (66.1, 100.0)	55.9 (0.0, 100.0)	**< 0.001**

Data are presented as median (Q1, Q3) or *N* (%). *p* values reference comparisons of the MMC vs. NICU recovery cohorts using Kruskal–Wallis tests for continuous variables and chi-squared test for sex. *p* values < 0.05 are in bold. The NICU recovery cohort combines participants from the NICU acoustics and technology development studies. One MMC cohort infant did not contribute supine time data

*MMC* myelomeningocele, *NICU* neonatal intensive care unit, *TST* total sleep time

### Distribution of sleep positions

In the NICU recovery cohort, infants spent more time (*p* < 0.001) sleeping supine (426.2 min, IQR 306.9–542.8) than nonsupine (36 min, IQR 0.0–175) (Table 3). In contrast, MMC cohort participants on the whole spent significantly less time (*p* < 0.001) in supine sleep (61.0 min, IQR 0–255.6). At many of the MMC study sites, infants with postnatal surgery were always placed in lateral or prone position to avoid pressure on the surgical site; however, this was not universal. Positioning for infants in the fetal surgery group generally included more time supine than the postnatal surgery group (Table 5, *p* = 0.005). Within the MMC cohort, the postnatal surgery group spent more time (*p* < 0.001) sleeping nonsupine (291 min, IQR 99–396.2) than supine (2.5 min, IQR 0–145.5) whereas in the fetal surgery groups, the time spent sleeping nonsupine (211 min, IQR 29.5–382.6) was not statistically different (*p* = 0.13) from that spent in supine sleep (122 min, IQR 0–293.6). The percentage of sleep time spent in supine sleep by each cohort is represented in the supplement (Supplementary Table S2).

**Table 3:** Sleep time and AHI by cohort and position for 261 neonates with polysomnograms

Variable	MMC (*N* = 173)	NICU recovery (*N* = 88)	*p* value	*p* value adjusted for gestational age
All sleep positions	*N* = 167	*N* = 88		
Percent of total sleep time in supine position	22.7 (0.0, 69.4)	93.4 (66.1, 100.0)	**< 0.001**	**< 0.001**
AHI	19.3 (10.0, 39.3)	19.8 (12.1, 36.4)	0.42	0.66
OAI	1.2 (0.5, 3.3)	2.2 (0.9, 4.1)	0.067	0.87
CAI	4.0 (1.2, 8.0)	6.3 (2.7, 11.6)	**0.006**	0.21
HI	10.9 (5.1, 21.4)	11.5 (5.5, 19.0)	0.66	0.76
Supine	*N* = 87	*N* = 87		
AHI	17.5 (6.2, 34.6)	20.2 (12.0, 36.6)	**0.032**	0.12
Nonsupine	*N* = 133	*N* = 39		
AHI	20.8 (11.3, 40.2)	20.2 (11.0, 38.3)	0.84	0.19

Data are presented as median (Q1, Q3). *p* values’ reference comparison of MMC vs. NICU recovery cohorts within each sleep position stratum (Kruskal–Wallis tests); “adjusted for gestational age” values are from log-AHI regression with GA as covariate. *p* values < 0.05 are highlighted in bold

*AHI* apnea–hypopnea index, *CAI* central apnea index, *GA* gestational age, *HI* hypopnea index, *MMC* myelomeningocele, *NICU* neonatal intensive care unit, *OAI* obstructive apnea index

### Sleep apnea severity by sleep position, adjusted for gestational age (GA)

The overall AHI was higher (*p* = 0.03) in the NICU recovery cohort (20.2, IQR 12–36.6) than the MMC cohort (17.5, IQR 6.2–34.6) in the unadjusted model, but there was no difference after adjustment for gestational age at birth (*p* = 0.10). For the NICU recovery cohort, the median AHI was 20.2 in both supine (IQR 12.0–36.6) and nonsupine (IQR 11–38.3) positions (*p* = 0.30). However, the impact of sleep position—supine vs. nonsupine—on AHI varied by MMC participant group (*p* < 0.001 for the interaction). Position-stratified medians for AHI, OAI, CAI, and HI (hypopnea index) are shown for the MMC cohort overall in Table 4, by surgical repair timing (group) in Table 5, and within the prenatal repair group (open fetal vs. fetoscopic surgery) in Table 6.

**Table 4:** Sleep indices in the neonatal myelomeningocele cohort by sleep position (*N* = 173)

Variable	Supine (*N* = 87)	Nonsupine (*N* = 133)	All positions (*N* = 167)	*p* value (supine vs. nonsupine)	*p* value adjusted for gestational age
AHI	17.5 (6.2, 34.6)	20.8 (11.3, 40.2)	19.3 (10.0, 39.3)	**0.010**	**0.001**
OAI	1.3 (0.5, 3.0)	1.3 (0.4, 3.2)	1.2 (0.5, 3.3)	0.51	0.83
CAI	4.6 (1.8, 8.4)	4.0 (1.2, 8.2)	4.0 (1.2, 8.0)	0.41	0.25
HI	8.4 (0.0, 21.0)	12.9 (6.9, 22.9)	10.9 (5.1, 21.4)	** < 0.001**	** < 0.001**

Data are presented as median (Q1, Q3). Within-group *p* values compare supine versus nonsupine sleep position (Kruskal–Wallis tests, unadjusted). Reported *p* values are unadjusted for gestational age; however, gestational age-adjusted analyses yielded the same conclusions and were congruent in significance, with equal or lower *p* values than the corresponding unadjusted analyses. *p* values < 0.05 are shown in bold

*AHI* apnea–hypopnea index, *CAI* central apnea index, *GA* gestational age, *HI* hypopnea index, *MMC* myelomeningocele, *OAI* obstructive apnea index

**Table 5:** Sleep indices in the neonatal myelomeningocele cohort by surgical repair timing: postnatal (*N* = 83) vs. fetal surgery (*N* = 90)

	Postnatal MMC repair (*N* = 83)			Fetal MMC repair, combined (*N* = 90)		*p* value
% of total sleep time in supine position	0.9 (0.0, 43.8)			37.9 (0.0, 90.4)		0.005
Variable	Supine (*N* = 33)	Nonsupine (*N* = 69)	*p* value	Supine (*N* = 54)	Nonsupine (*N* = 64)	*p* value
AHI	15.8 (6.2, 25.7)	18.9 (10.6, 34.5)	0.28	19.4 (6.3, 38.1)	23.1 (12.1, 45.1)	**0.011**
OAI	1.3 (0.2, 2.5)	0.8 (0.2, 2.3)	0.061	1.3 (0.6, 3.1)	1.8 (0.5, 5.2)	0.56
CAI	4.6 (1.7, 7.9)	3.8 (1.0, 7.2)	0.98	4.3 (1.8, 8.4)	4.5 (1.3, 10.2)	0.24
HI	6.7 (0.5, 14.7)	12.6 (7.0, 19.6)	**0.002**	8.6 (0.0, 22.6)	15.1 (6.9, 27.3)	** < 0.001**

Data are presented as median (Q1, Q3). Within-group *p* values compare supine versus nonsupine sleep position (Kruskal–Wallis tests, unadjusted). Reported *p* values are unadjusted for gestational age; however, gestational age-adjusted analyses yielded the same conclusions and were congruent in significance, with equal or lower *p* values than the corresponding unadjusted analyses. *p* values < 0.05 are shown in bold. The prenatal MMC repair group combines the fetoscopic repair group and the open fetal repair group

*AHI* apnea–hypopnea index, *CAI* central apnea index, *GA* gestational age, *HI* hypopnea index, *MMC* myelomeningocele, *OAI* obstructive apnea index

**Table 6:** Sleep indices across the prenatal MMC repair groups: open fetal surgery (*N* = 56) vs. fetoscopic surgery (*N* = 34), by sleep position

	Open fetal surgery (*N* = 56)			Fetoscopic surgery (*N* = 34)		*p* value
% of total sleep time in supine position	46.8 (0.0, 93.6)			31.5 (0.0, 67.0)		0.47
Variable	Supine (*N* = 33)	Nonsupine (*N* = 39)	*p* value	Supine (*N* = 21)	Nonsupine (*N* = 25)	*p* value
AHI	29.5 (13.7, 55.9)	25.9 (17.0, 44.7)	0.76	6.3 (3.5, 24.2)	22.9 (7.5, 46.3)	** < 0.001**
OAI	1.3 (0.3, 3.4)	1.8 (0.4, 4.8)	0.62	1.7 (0.7, 3.1)	1.6 (0.6, 5.4)	0.61
CAI	4.9 (1.8, 8.4)	5.8 (1.8, 9.6)	0.57	3.1 (0.6, 8.4)	4.5 (0.7, 11.5)	0.19
HI	18.0 (8.4, 35.0)	16.0 (9.9, 27.7)	0.48	0.0 (0.0, 0.0)	10.4 (4.6, 25.7)	** < 0.001**

Data are presented as median (Q1, Q3). Within-group *p* values compare supine versus nonsupine sleep position (Kruskal–Wallis tests, unadjusted). Reported *p* values are unadjusted for gestational age; however, gestational age-adjusted analyses yielded the same conclusions and were congruent in significance, with equal or lower *p* values than the corresponding unadjusted analyses. *p* values < 0.05 are shown in bold. The fetoscopic repair group shows a significantly lower AHI in the supine position, driven primarily by the HI

*AHI* apnea–hypopnea index, *CAI* central apnea index, *GA* gestational age, *HI* hypopnea index, *MMC* myelomeningocele, *OAI* obstructive apnea index

**Fig. 1:**
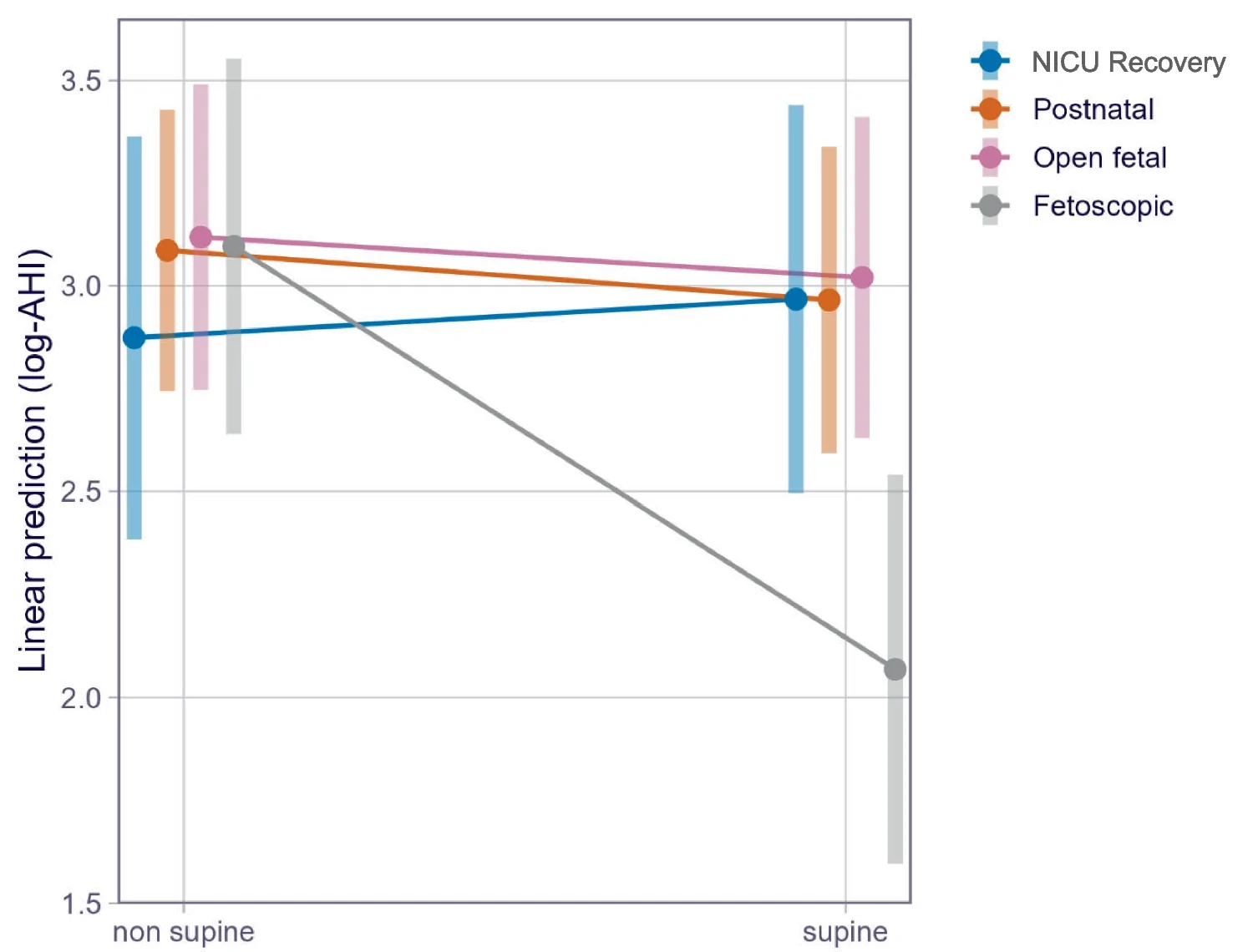
Estimated log-AHI by sleep position and participant group for 261 neonates. Linear-prediction (log-AHI) estimates with 95% confidence intervals from the linear mixed model described in Table 7, plotted by sleep position (nonsupine vs. supine) for each of the four participant groups. Note the marked separation of the fetoscopic group in the supine position relative to the other three groups and the near-parallel lines for the NICU recovery, postnatal, and open fetal myelomeningocele groups, illustrating the position × group interaction

In adjusted analyses, the AHI was similar between supine and nonsupine sleep positions in the NICU recovery, postnatal repair MMC, and open fetal repair MMC groups (Table 7, Fig. 1). In contrast, infants in the fetoscopic repair MMC group had substantially lower AHI (*p* < 0.001) when sleeping supine (estimated geometric mean 7.94, 95% CI 4.95–12.75) compared to nonsupine (22.15, 95% CI 14.02–35.01). In the supine position, the fetoscopic repair MMC group had significantly lower AHI than each of the three other groups (*p* < 0.004 for each pairwise comparison). No other position-related differences were observed across groups (all other *p* > 0.33).

**Table 7:** AHI by sleep position and group for newborns in the neonatal intensive care unit (linear mixed model, adjusted for gestational age)

Participant group	Nonsupine AHI (mean, 95% CI)	Supine AHI (mean, 95% CI)
NICU recovery (*N* = 88)	17.7 (10.9, 28.9)	19.5 (12.1, 31.2)
Postnatal MMC surgery (*N* = 79)	21.9 (15.6, 30.9)	19.4 (13.4, 28.2)
Open fetal MMC surgery (*N* = 55)	22.6 (15.6, 32.8)	20.5 (13.9, 30.3)
Fetoscopic MMC surgery (*N* = 33)	22.1 (14.0, 34.9)	*7.9 (4.9, 12.7)

Estimated geometric mean AHI (95% CI), back-transformed from a linear mixed model fit on log(AHI) with fixed effects for sleep position, participant group, their interaction, and gestational age, and random intercepts for participant and site (*N* = 346 observations from 255 participants). The interaction term for group by sleep position was overall statistically significant (*p* < 0.001). Pairwise differences between groups and positions were then tested

*Statistically significant differences between fetoscopic MMC and other groups in supine AHI (*p* < 0.004 for each comparison to the three other supine groups), but no other differences between groups in AHI were significant for supine or nonsupine positions

*MMC* myelomeningocele

The AHI positional difference within the fetoscopic MMC group (*p* < 0.001) was driven primarily by the hypopnea index, which was lower supine (median 0.0, IQR 0–0) than nonsupine (median 10.4, IQR 4.6–25.7). No within-group differences emerged for the obstructive apnea index (OAI) or central apnea index (CAI) by position.

### Sensitivity analyses

The lower supine AHI within the fetoscopic MMC group persisted in sensitivity analyses. The primary linear mixed model accounted for between-site variation through a random intercept at the site level (Table 7); a model that excluded data more than three standard deviations from the within-group mean log-AHI demonstrated similar results (*p* < 0.001). Sensitivity analyses using a range of inclusion thresholds for TST per sleep position showed that the lower supine AHI in the fetoscopic MMC group was present using a minimum sleep threshold of 30 min (strongest effect) and persisted at the primary 60-min threshold. The trend remained at 120 min, but statistical significance was lost, consistent with reduced power as sample sizes decreased. Full sensitivity analysis results are provided in the supplementary material (Supplementary Table S3).

## Discussion

In this detailed analysis of polysomnographic data from 261 neonates who required neonatal intensive care due to myelomeningocele or other conditions such as neonatal respiratory failure or prematurity, virtually no consistent or clinically meaningful variation in AHI between supine and nonsupine sleep positions emerged. The overall lack of difference in AHI by sleep position in most of the infants differed from the typical patterns seen in older children and adults, who often have obstructive and central sleep apnea that is worse in supine than nonsupine positions [3–5, 15–17]. In the one MMC subgroup with positional variation in sleep apnea (fetoscopic repair), the position effect was in the opposite direction of the typical pattern in older children or adults. The tendency for sleep apnea to be worse on average when supine is so often reported for older children and adults that the apparent absence of this pattern in neonates is remarkable.

In older children and adults, AHI is consistently higher on average in supine sleep compared to nonsupine sleep [3–5, 13–15]. The factors that protect neonates from positional sleep apnea are not yet clear, but further understanding may also illuminate the mechanisms that produce this phenomenon at older ages. One possibility derives from the observation that a brief stimulus of a droplet in the upper airway—by extrapolation, not much different from brief obstruction that is more likely to occur supine—triggers central apnea. This was demonstrated many years ago in lamb models [16–18]. Yet, data on positional central sleep apnea remain limited in children, for whom mechanisms for central sleep apnea may be different from adults.

Some prior studies of infants help contextualize our findings. A study of 347 infants at high risk for SIDS [6] examined the frequency of extreme cardiorespiratory events (prolonged apnea or significant bradycardia), using home monitoring as described in the original CHIME study [19]. Infants placed in nonsupine positions were *not* more likely to experience extreme cardiorespiratory events than those in the supine position, suggesting that the lower SIDS frequency with supine sleep is achieved by mechanisms other than cardiorespiratory events detectable by the home monitors.

Data regarding sleep position effects on sleep apnea in older infants are limited and have led to mixed conclusions. Similar to our findings, a study of 64 infants [20] reported a lack of central or obstructive apnea (or sleep state) dependence on position, though scoring criteria were markedly different at the time of that publication in 1985. Furthermore, in a 2008 study of 50 babies 8–12 months of age [21], no significant difference emerged between supine AHI and nonsupine AHI.

In contrast, a more recent study of 4-h polysomnograms among 72 infants less than 6 months of age who did not have central breathing disorders (median sleep duration 2.4 h) reported that supine position was associated with more obstructive respiratory events with an obstructive AHI (O-AHI) of 8 in the supine position compared to O-AHI 4 in the lateral position [22]. However, in that study, central apneas had the reverse direction of position dependence: there was a statistically higher central apnea index (CAI) in the lateral position (9) compared to the supine position (6) [22]. This study differed from ours in that it was a 10-year retrospective review of clinically indicated polysomnograms (35% of which were performed due to acute life-threatening event history or sibling with SIDS), selected for specific sleep-disordered breathing results, and did not include syndromic infants. In a different study [23], prone positioning in 24 very premature infants (born significantly more premature than our cohort with a mean gestational age of 27.9 weeks) was associated with higher frequency of central respiratory events compared to supine positioning; lateral sleep was not studied. In contrast to our participants, over half of that study group also had bronchopulmonary dysplasia and the infants were treated with supplemental oxygen.

The mechanism for the general lack of AHI dependence on position in our neonatal cohorts—in contrast to previous evidence from children and adults—is uncertain. We speculate that obligate nasal breathing may provide protective mechanisms against gravity-related obstructive events, such as supine retrusion of the mandible and mouth breathing, in older children. Further, adenotonsillar hypertrophy is not routinely present in newborn infants [24] as it is in older children. Thus, this common pediatric obstructive sleep apnea etiology, which may be positional, would not be expected in our cohort of newborn infants. Our study’s participants have a higher frequency of central than obstructive respiratory events. Whether central sleep apnea has a clear position dependence in children does not appear to be addressed in our review of published literature.

We do not have a clear mechanistic explanation for the observation that the infants with MMC treated with fetoscopic surgery (compared to open fetal or postnatal surgery, which have very similar infant characteristics) would have a lower supine AHI than lateral AHI, although the effect persisted despite careful sensitivity analyses. This could be a spurious finding due to limited sample size for the fetoscopic surgery group. It is unclear whether some difference may exist in the postsurgical airway of this group (perhaps different respiratory mechanics postdelivery or different brainstem outcomes affecting the respiratory drive). We consider the effects of cerebral spinal fluid (CSF) dynamics and of respiration, on both CSF pressure and chemoreceptor mechanisms [25–27], as possible pathways to positional apnea: this may affect all infants but particularly infants who have altered CSF flow due to myelomeningocele. However, such impact may not explain the surgical group differences we noted, as fetoscopic and open fetal repair groups would likely be subject to similar physiology. Notably, infants with myelomeningocele who underwent fetoscopic surgery remain understudied as fetoscopic surgery is a relatively new technique. We have higher confidence in the absence of a difference in AHI between supine and nonsupine sleep positions in the remainder of the groups (nonfetoscopic surgical MMC groups and NICU recovery cohort), due to higher sample sizes.

It is worth noting that the MMC study population did not show worse sleep apnea severity than the predominantly preterm NICU recovery cohort, despite myelomeningocele being an established risk factor for neonatal sleep apnea [28]. Once gestational age was accounted for, AHI was statistically similar between the two cohorts. This suggests that prematurity itself (or other critical illness in the neonatal period), with its associated immature respiratory control, contributes substantially to neonatal sleep apnea severity and may approach that seen in myelomeningocele.

Strengths of this study include the use of gold-standard polysomnographic data and relatively high sample size. These analyses were based on approximately 2786 cumulative hours (116 days) of full, attended sleep studies, which captured about 1901.5 h of neonatal sleep. We were able to study two types of NICU populations, one without many major comorbidities (predominantly convalescing premature infants without other risk factors) and the other with neurodevelopmental disabilities as a result of myelomeningocele, a known risk factor for sleep apnea in infancy [28]. The effects of prematurity may influence polysomnographic outcomes [29, 30], so we adjusted analyses for gestational age.

Limitations of this study include the secondary analysis design. Our data set did not include AHI values specific to sleep stages (REM vs. NREM sleep). In the MMC cohort, variations by study center in sleep positioning were significant. Prone sleep was rare and concentrated at a single center, precluding a separate analysis of prone-versus-supine effects; prone observations were grouped with nonsupine in the primary analyses. The data reported here are polysomnographic findings in the hospital setting and may not apply to home or other “real-world” settings.

Because sleep position was not randomized but was determined by clinical and postsurgical care patterns, residual confounding between sleep position and underlying medical condition cannot be fully excluded. However, the consistent absence of position dependence across both the MMC and NICU recovery cohorts—with quite different underlying conditions—argues against a single confounder driving the result. Our study could not measure CSF pressure variations in order to directly test whether position-dependent CSF redistribution mediates the observed positional AHI differences. We do not believe that body fluid shifts documented in older patients, who spend the day upright and the night supine, are likely to have impact on the sleep-disordered breathing of newborns, who do not spend appreciable time upright. We also did not collect detailed information on the etiology of preterm birth or other reasons for NICU admission in the NICU acoustics and technology development studies, which limits inference about whether specific perinatal factors influence positional sleep apnea. Finally, obstructive and central apnea subindices were not available by position in the NICU recovery cohort’s source datasets, so OAI/CAI position comparisons could be reported only for the MMC cohort.

## Conclusions

In this study of 261 neonates who underwent gold-standard polysomnography in the hospital setting, no meaningful variation was generally found in AHI between supine and nonsupine positions. The one notable exception to this was the small subgroup of infants who underwent fetoscopic myelomeningocele repair, for whom supine sleep was associated with markedly lower AHI. The overall lack of position dependence in this study is in distinct contrast to what is reported across the rest of the human lifespan, during which sleep apnea is typically worse with supine positioning. Understanding the mechanisms that protect neonates from positional sleep apnea—we speculate factors related to obligate nasal breathing—could illuminate protective pathways relevant to sleep apnea at older ages. For example, perhaps better preservation of nasal breathing throughout the lifespan could help avoid development of sleep apnea. Practically, these data also support the reliability of polysomnographic data obtained during nonsupine sleep for most infants in the NICU—in other words, that assessment in nonsupine or supine sleep may yield similar results—allowing evaluation before an infant is consistently positioned supine. Finally, results of the present analyses may serve to allay concerns that “Back to Sleep”—a necessary life-saving intervention for infants upon discharge to home—could at the same time worsen sleep apnea.

## Supplementary Information

Below is the link to the electronic supplementary material.

ESM 1 (DOCX 22.0 KB)

## Data Availability

All data supporting the findings of this study are available within the paper and its supplementary information.
