# Supplementary material for: Sleep position and sleep apnea in newborns
Source: J Clin Sleep Med. 2026 Nov 3;22(1):211. doi: 10.1007/s44470-026-00218-0 (PMC13731558; doi:10.1007/s44470-026-00218-0)
Supplement: Supplementary file 1 [file 44470_2026_218_MOESM1_ESM.docx]

**Supplementary Data**

**Table S1: Variation of sleep position by study site for 173 neonates with myelomeningocele**

| Variable | **01 (N=19)** | **02 (N=8)** | **03 (N=25)** | **04 (N=21)** | **05 (N=44)** | **06 (N=6)** | **07 (N=4)** | **08 (N=32)** | **09 (N=14)** | p value |
| --- | --- | --- | --- | --- | --- | --- | --- | --- | --- | --- |
| Age* (wk) NICU PSG |  |  |  |  |  |  |  |  |  | **<0.001** |
| Median (Q1,Q3) | 0.7 (0.4, 1.9) | 1.6 (1.1, 2.9) | 0.7 (0.4, 0.9) | 2.7 (2.0, 4.0) | 1.3 (0.9, 2.0) | 2.1 (0.8, 3.0) | 3.8 (2.0, 5.6) | 2.1 (1.6, 3.0) | 1.5 (1.3, 2.2) |  |
| Mean (SD) | 1.1 (0.8) | 2.4 (2.2) | 0.8 (0.7) | 3.3 (2.3) | 1.8 (1.7) | 2.1 (1.6) | 3.8 (2.2) | 3.0 (2.3) | 1.8 (0.9) |  |
| Supine time/TST (%) |  |  |  |  |  |  |  |  |  | **<0.001** |
| Median (Q1, Q3) | 1.6 (0.0, 37.8) | 87.5 (0.0, 100.0) | 0.0 (0.0, 0.0) | 37.0 (0.0, 74.2) | 57.1 (0.0, 100.0) | 7.8 (0.2, 27.1) | 65.7 (23.5, 100.0) | 42.2 (6.5, 68.8) | 0.0 (0.0, 18.4) |  |
| Mean SD) | 22.5 (34.2) | 59.4 (49.9) | 2.0 (5.9) | 43.7 (39.8) | 52.3 (41.0) | 13.9 (16.6) | 57.8 (50.3) | 43.7 (36.3) | 20.6 (38.0) |  |

*Age is postmenstrual age at the time of the polysomnogram

**Table S2: Supine sleep time as a percentage of total sleep time, by participant group**

| **Participant group** | **N** | **Supine time / TST (%), Median (Q1, Q3)** |
| --- | --- | --- |
| **Postnatal MMC** | 83 | 0.9 (0.0, 43.8) |
| **Open Fetal MMC** | 56 | 46.8 (0.0, 93.6) |
| **Fetoscopic MMC** | 34 | 31.5 (0.0, 67.0) |
| **NICU recovery** | 88 | 93.4 (66.1, 100.0) |

*Notes and abbreviations:* MMC = Myelomeningocele; TST = total sleep time. Percent of total sleep time (TST) spent in the supine position, by participant group. The marked variation in supine positioning across the MMC subgroups reflects differences in post-surgical care: postnatal repair infants are largely kept non-supine to protect the surgical site, whereas fetal-repair infants (open and fetoscopic) tolerate more supine positioning.

**Table S3: Sensitivity Analysis - Linear Mixed Model of outcome log-AHI**

| **Linear Mixed Model of outcome log-AHI** | | | |
| --- | --- | --- | --- |
|  | | | |
| Model Results | Exp(beta) | 95% confidence interval | p-value |
| Supine (vs non supine) | 1.10 | (0.90,1.34) | 0.374 |
| Estimated gestational age | 0.92 | (0.87,0.96) | <0.001*** |
| Postnatal (vs preterm) | 1.20 | (0.77,1.86) | 0.422 |
| Open fetal (vs preterm) | 1.26 | (0.82,1.97) | 0.314 |
| Fetoscopic (vs preterm) | 1.24 | (0.70,2.14) | 0.463 |
| Supine*Postnatal interaction | 0.81 | (0.59,1.10) | 0.191 |
| Supine*Open fetal interaction | 0.83 | (0.59,1.16) | 0.281 |
| Supine*Fetoscopic interaction | 0.33 | (0.22,0.48) | <0.001*** |
| ------ |  |  |  |
| variance components: |  |  |  |
| study_id\|(Intercept) SD | 0.678 |  |  |
| site_comb\|(Intercept) SD | 0.311 |  |  |
| Residual SD | 0.469 |  |  |
| --- |  |  |  |
| N | 346 |  |  |
| AIC | 817.76 |  |  |
